# Accuracy of ^68^Ga-PSMA PET-CT and PET-MRI in lymph node staging for localized prostate cancer

**DOI:** 10.31744/einstein_journal/2022AO6599

**Published:** 2022-05-06

**Authors:** Álan Roger Gomes Barbosa, Breno Santos Amaral, Danilo Budib Lourenço, Bianca Bianco, Felipe Arakaki Gushiken, Marcelo Apezzato, Júlia Francisco Silva, Marcelo Livorsi da Cunha, Reneé Zon Filippi, Ronaldo Hueb Baroni, Gustavo Caserta Lemos, Arie Carneiro

**Affiliations:** 1 Hospital Israelita Albert Einstein São Paulo SP Brazil Hospital Israelita Albert Einstein, São Paulo, SP, Brazil.; 2 Centro Universitário FMABC Santo André SP Brazil Centro Universitário FMABC, Santo André, SP, Brazil.; 3 Faculdade Israelita de Ciências da Saúde Albert Einstein Hospital Israelita Albert Einstein São Paulo SP Brazil Faculdade Israelita de Ciências da Saúde Albert Einstein, Hospital Israelita Albert Einstein, São Paulo, SP, Brazil.

**Keywords:** Prostatic neoplasms, Lymph nodes, Prostatectomy, Data accuracy, Positron emission tomography computed tomography, Prostate-specific antigen, Gallium

## Abstract

**Objective:**

To evaluate the predictive value of positron emission computed tomography or magnetic resonance (PET-CT and PET-MRI) using gallium-68-labeled prostate-specific membrane antigen (^68^Ga-PSMA) in lymph node involvement in prostate cancer.

**Methods:**

A retrospective study comprising 91 patients diagnosed with prostate cancer between 2016 to 2020, who underwent ^68^Ga-PSMA PET-CT or PET-MRI for staging before prostatectomy. The patients were divided into Group 1, with 65 patients with satisfactory pathological lymph node analysis, and Group 2, with 91 patients representing the sum of patients with pathological lymph node analysis and those with postoperative prostate-specific antigen within 60 days after surgery. Receiver Operating Characteristic curves were used to assess accuracy of predictive capacity of imaging exams for lymph node involvement.

**Results:**

Regarding local clinical staging, the groups showed similar results, and 50% were classified as staging T2a. The accuracy of ^68^Ga-PSMA PET-CT for prostate cancer lymph node staging was 86.5% (95%CI 0.74-0.94; p=0.06), with a sensitivity of 58.3% and specificity of 95%. The accuracy of ^68^Ga-PSMA PET-MRI was 84.6% (95%CI 0.69-0.94; p=0.09), with a sensitivity of 40% and specificity of 100%. Considering both ^68^Ga-PSMA PET-CT and PET-MRI, the accuracy was 85.7% (95%CI 0.76-0.92; p=0.015), with sensitivity of 50% and specificity of 97%.

**Conclusion:**

The imaging tests ^68^Ga-PSMA PET-CT and PET-MRI were highly accurate to detect preoperative lymph node involvement, and could be useful tools to indicate the need for extended lymph node dissection during radical prostatectomy.

## INTRODUCTION

Prostate cancer is the most prevalent solid tumor among men. It is estimated that approximately 61,200 cases are diagnosed annually in Brazil, accounting to roughly 23% of all cancers diagnosed in men in the country. Of these cases, approximately 12,000 were diagnosed in the state of São Paulo.^(1)^

Pelvic lymph nodes are a common site of recurrent disease and may change the clinical management. Using conventional imaging tests, it is still a challenge to detect the exact site of recurrence and optimally guide personalized treatment.^(2,3)^ In men treated with radical prostatectomy for localized prostate cancer, the presence of lymph node metastases is a strong adverse prognostic factor associated with higher recurrence rates and shorter long-term survival.^(4)^ Accurate nodal staging (N staging) is therefore key for planning treatment and postoperative follow-up.^(5)^ It is estimated that approximately 40% of high-risk lesions initially staged as no node metastasis (N0) actually have lymph node metastases.^(6,7)^

Positron emission tomography (PET) is a tomographic technique that measures, in three dimensions, the distribution of positron-emitting radiopharmaceuticals (a drug that has a radioactive element in its composition), which allows non-invasive quantitative and qualitative assessment of physiological and biochemical processes.^(8)^ Positron emission computed tomography (PET-CT) and magnetic resonance imaging (PET-MRI) techniques have been widely used to detect the presence of lymph nodes metastases, but none of these methods proved to be accurate for preoperative N staging.^(9)^

New technologies have been studied aiming to increase diagnostic accuracy, with special focus on nuclear medicine and new radiopharmaceuticals. Prostate membrane-specific antigen (PMSA) is a cell membrane-binding protein that is overexpressed in malignant prostate cancers, especially adenocarcinoma and its metastases, and is found at low levels in healthy organs. In the case of prostate cancer, the expression of this protein tends to be associated with tumor differentiation.^(10)^ The radiopharmaceutical labeled with gallium 68, ^68^Ga-PSMA-11-HBED-CC, or simply ^68^Ga-PSMA, is a synthetic antigen inhibitor that, when injected into the patient, binds to the cells of the primary prostate cancer and its metastases. Recent studies show encouraging results from^68^Ga-PSMA PET-CT in patients with biochemical relapse, with greater accuracy for lymph node evaluation when compared to conventional exams;^(11,12)^ and the use of ^68^Ga-PSMA PET-CT has already become a recommendation in guidelines for investigation of patients with biochemical recurrence. Freitag et al.^(10)^ reported the overall disagreement in PET-positive findings between ^68^Ga-PSMA PET-CT and PET-MRI was very low, and the consistence between the methods was very high.

To this day, the gold standard for node staging still is extended dissection of pelvic nodes. The decision for node dissection must be guided according to the preoperative probability of nodal metastases,^(9,13)^ since this surgical procedure is associated to increased morbidity and higher complications rates.^(14)^

## OBJECTIVE

To evaluate the accuracy of ^68^Ga-PSMA PET-CT and PET-MRI in localized prostate cancer lymph node staging, besides the unfavorable intermediate and high-risk subgroups, and local staging.

## METHODS

### Patients

An observational, retrospective, cohort study that included 91 patients diagnosed as prostate cancer, who underwent radical prostatectomy with extended lymph node dissection, and were evaluated with preoperative ^68^Ga-PSMA PET-CT or PET-MRI, in the period of January 2016 to July 2020. Patients who underwent preoperative radiotherapy and/or hormone blockade were excluded.

Data collected from the patients’ medical records included age upon surgery (years), pre-biopsy and postoperative prostatic specific antigen (PSA) (ng/mL), TNM staging (T for tumor, N for lymph node and M for metastasis), Gleason score, number of affected fragments, prostate volume (cc), ^68^Ga-PSMA PET-CT or PET-MRI results (lymph node involvement, TNM staging or maximum standardized uptake value - SUV), pathological result according to the International Society of Urological Pathology (ISUP), or number of affected lymph nodes and specimen tumor staging.

The project was approved by the Ethics Committee of *Hospital Israelita Albert Einstein* (HIAE), # 2,558,767, CAAE: 83527518.4.0000.0071. Signing the Informed Consent Form was waived by the Research Ethics Committee.

### Analysis of 68Ga-PSMA PET-CT and PET-MRI

All studies were initiated 50±5 minutes after the intravenous administration of a single dose of ^68^Ga-PSMA (mean injected activity of 195 Megabecquerel – MBq –, always aiming to administer at least 2.0 MBq/kg of body weight). ^68^Ga-PSMA was produced by the Radiopharmaceutical Division of the Nuclear Medicine Department at the HIAE, according to a standardized and previously established protocol.^(15)^

The ^68^Ga-PSMA PET-CT data were acquired on a dedicated Biograph Scanner mCT PET-CT device (32-slice CT; Siemens Medical Solutions, Erlangen, Germany). Images from the vertex of the skull to the proximal half of the thighs were obtained, with the patients with their arms raised, with 4 minutes of acquisition per evaluated segment (bed position). The obtained images were reconstructed using the ultra-HD method (TrueX and time of flight) with two iterations, 21 subsets, 200×200 matrix, and using a 3mm Gaussian filter with attenuation correction based on CT (100kV, 156mAs, 1.5 pitch, and rotation time of 0.5 second).

Data from the whole-body ^68^Ga-PSMA PET-MRI were acquired in a hybrid PET Biograph Scanner mMR (Siemens Medical Solutions) equipment, with images from the vertex of the skull to the proximal half of the thighs, upper limbs positioned alongside the trunk, with 4 minutes of acquisition per evaluated segment (bed position). A 24-channel column radiofrequency coil integrated inside the equipment stretcher and three surface body coils (six channels each) were used to include the chest, abdomen and pelvis. For the neck, an 8-channel radiofrequency head/neck coil was used. Simultaneously with the acquisition of the PET images, the data for the magnetic resonance images were obtained, performing axial T1-weighted (VIBE) Dixon and multiplanar T2-weighted sequences, before and after the administration of paramagnetic contrast. Images were reconstructed using a 3D-OSEM algorithm (two iterations, 21 subsets, 256×256 matrix, using a 4mm Gaussian filter) and corrected for attenuation using Dixon-based resonance sequences.

For image analysis, a dedicated workstation equipped with syngo.via software (Siemens Medical Solutions) was used.

Images were reviewed by a nuclear medicine physician and a radiologist, both experienced in PET-CT and PET-MRI.

Areas of anomalous ^68^Ga-PSMA uptake (different from the usual and physiological biodistribution) in PET-CT or PET-MRI studies, with corresponding abnormalities in structural PET-CT or PET-MRI images, were considered positive for lesions related to prostate cancer. Quantitative analyses of the lesions were established by calculating the maximum SUV for each of them. The standardized volumes of interest were defined by the physician responsible for reading the images, and the value obtained by automatic calculation using the syngo.via^®^ software, was recorded.

Doubtful cases or disagreements between examiners were solved by consensus. All pieces of clinical information, including laboratory and other imaging method data, whenever available, were considered in interpretation of the studies.

The following data were analyzed: date of examination, result of the analysis of lymph node status (involved or not secondary), number of lymph nodes when involved, presence of extra prostatic extension, invasion of seminal vesicles or other organs, prostate size and maximum SUV.

### Histological analysis of primary tumor and pelvic lymph nodes

The slides were reviewed by pathologists with no access to clinical data or direct patient identification. The results according to International Society of Urological Pathology ( ISUP) and the description of any pathological abnormalities in the prostatic zones were analyzed. The lymph nodes were assessed by pathologists and considered as positive or negative for secondary involvement, besides the total number of nodes seen in the sample and the total number with disease.

### Analysis of accuracies

After these analyses, accuracy tests of ^68^Ga-PSMA PET-CT or PET-MRI were conducted, comparing the preoperative results and the pathological reports.

To analyze the accuracy of ^68^Ga-PSMA PET-CT or PET-MRI, PET was considered negative for lymph node disease when the report did not show radiologically suspicious lymph nodes and ^68^Ga-PSMA uptake. Patients with no lymph node disease underwent extended lymph node dissection (pathological report with 12 or more lymph nodes resected), with no lymph node disease in the pathological evaluation, and PSA <0.03ng/mL within 60 days of the procedure. For sub-analysis, patients with no lymph node disease were considered those who did not undergo extended lymph node dissection, but had PSA <0.03ng/mL within 60 days of the procedure.

### Statistical analysis

Statistical analysis was carried out using the R programming language (RStudio version 1.2.5042).^(16)^ Data distribution normality was assessed using the Shapiro-Wilk test. Student’s *t* test was used to test groups of continuous variables with normal distribution. Continuous data with no normal distribution were compared using the Mann-Whitney U test.

As a performance metric, the Area Under the Curve (AUC) Analysis of the Receptor Operating Characteristic (ROC) was conducted to assess the accuracy of ^68^Ga-PSMA PET-CT and PET-MRI in predicting lymph node involvement in prostate cancer .

Binomial logistic regression analysis was carried out to test the association between lymph node invasion on pathological examination and preoperative variables. The dependent variable of the test was the categorical outcome of lymph node invasion on pathological examination (positive or negative), the independent variables being the continuous data on age and initial PSA, and categorical data on ^68^Ga-PSMA PET-CT and PET-MRI with preoperative detection of involved lymph nodes (positive or negative), ISUP and Gleason with their respective grades. Since the variables had more than two categories, it was necessary to transform them into dummy variables to accommodate them in the binomial model.^(17)^ Statistical significance was set at 5% or p value <0.05.

## RESULTS

Data from 226 patients who underwent ^68^Ga-PSMA PET-CT or PET-MR were analyzed. Of these patients, 89 were excluded for not having lymph node analysis in the imaging report, and 22 for not having a pathological report available for data collection. Of the 115 patients included in the study, 65 had satisfactory lymph node analysis on pathological examination (more than 11 resected lymph nodes); 26 did not present satisfactory lymph node analysis on pathological examination but had postoperative serum PSA within 60 days of surgery, and 24 patients did not fulfill these criteria and were also excluded from the study.

The patients included were divided into two groups: Group 1 had 65 patients with satisfactory lymph node analysis on pathological examination, and Group 2 had 91 patients representing the sum of those with satisfactory lymph node analysis on pathological examination and those with postoperative analysis of PSA within 60 days after surgery. The groups description is depicted in table 1.

**Table 1:** Characterization of the groups studied

Variables*	Group 1		Group 2	
	Total (n=65)	Unfavorable intermediate and high risk (n=38)	Total (n=91)	Unfavorable intermediate and high risk (n=51)
Age, years	66.0 (59.0-71.0)	67.0 (61.0-71.5)	65.0 (58.7-69.2)	66.0 (60.7-71.2)
BMI, kg/m^2^	27.0 (26.0-30.7)	27.0 (26.0-30.2)	27.0 (25.5-30.0)	27.0 (25.0-30.0)
PSA, ng/dL	6.0 (4.5-10.0)	6.1 (5.1-10.8)	5.5 (3.6-8.2)	5.9 (4.8-10.3)

Results expressed as median and 95% confidence interval.

BMI: body mass index; PSA: prostatic specific antigen.

There was no difference between age (p=0.517), body mass index (BMI) (p=0.535) and initial PSA (p=0.597) between groups. Considering the intermediate and high-risk subgroups in Groups 1 and 2, there was also no significant difference for age (p=0.931), BMI (p=0.569) and initial PSA (p=0.7545).

Regarding the local clinical staging (T), both Group 1 and Group 2 had similar results, and 50% were classified as stage T2a (Table 2).

**Table 2:** T clinical staging of the groups studied

Clinical staging	Group 1	Group 2
T2a	50	51
T2b	14	13
T2c	16	16.5
T3a	11	11.5
T3b	9	8

Results expressed as %.

The accuracy of ^68^Ga-PSMA PET-CT for lymph node staging of prostate cancer was 86.5% 95% confidence interval (95%CI) (95%CI 0.74-0.94; p=0.06), with sensitivity of 58.3% and specificity of 95%. The accuracy of ^68^Ga-PSMA PET-MRI was 84.6% (95%CI 0.69-0.94; p=0.09), with sensitivity of 40% and specificity of 100%. Adding up the analyses of both imaging exams (^68^Ga-PSMA PET-CT and PET-MRI), accuracy was 85.7% (95%CI 0.76-0.92; p=0.015), with sensitivity of 50% and specificity of 97% to identify lymph node involvement (Table 3).

**Table 3:** Accuracy of 68Ga-PSMA PET-CT and PET-MRI for prostate cancer lymph node staging

Variables	^68^Ga-PSMA PET-CT (n=52)	^68^Ga-PSMA PET-MRI (n=39)	^68^Ga-PSMA PET-CT + MRI (n=91)
Accuracy	86.5	84.6	85.7
95%CI	(0.74-0.94)	(0.69-0.94)	(0.76-0.92)
P value	0.06	0.09	0.015
Sensitivity	58.3	40	50
Specificity	95	100	97

Accuracy, sensibility, and specificity expressed as %.

MRI: magnetic resonance; 95%CI: 95% confidence interval.

Considering only the unfavorable intermediate and high-risk subgroup, according to D’Amico’s classification, and dividing the analyses into Group 1 (only pathological examination analysis compared to ^68^Ga-PSMA PET-CT or MRI), and Group 2 (comparing ^68^Ga-PSMA PET-CT or MRI with pathological results and postoperative PSA), the accuracy of the examination in Group 1 was 81.6% (95%CI 0.66-0.92; p=0.011), with sensitivity of 50% and specificity of 100%, and in Group 2, 84% (95%CI 0.71-0.93; p=0.018 ), with sensitivity of 46.7% and specificity of 100% (Table 4).

**Table 4:** Accuracy of 68Ga-PSMA PET-CT or PET-MRI for lymph node staging in unfavorable intermediate or high-risk patients, according to the D’Amico’s risk classification for prostate cancer

Variables	Group 1	Group 2
Accuracy	81.6	84
95%CI	(0.66-0.92)	(0.71-0.93)
P value	0.011	0.018
Sensitivity	50	46.7
Specificity	100	100

Accuracy, sensibility, and specificity expressed as %.

95%CI: 95% confidence interval.

Analyzing the accuracy of ^68^Ga-PSMA PET-CT or MRI for the detection of extra prostatic extension (compared with analysis of pathological examination), sensitivity was 10% for ^68^Ga-PSMA PET-CT and increased to 58% with ^68^Ga-PSMA PET-MRI, with specificity of 96.5% for PET-CT and 92.3% for ^68^Ga-PSMA PET-MRI. Improved sensitivity, when comparing ^68^Ga-PSMA PET-MRI to PET-CT, was also seen when the ability to detect seminal vesicle involvement (71.4% *versus* 40%), was analyzed – in that, 100% specificity for the former, and 95.5% for the latter.

The logistic regression analysis showed a significant association between the positive finding on ^68^Ga-PSMA PET-CT+PET-MRI, and the positive result of the pathological examination for lymph node involvement (p<0.001), in addition to an association between the initial PSA and the positive pathological result for lymph node involvement (p=0.018). No association was found between positive pathological results for lymph node involvement and age (p=0.88), type of PET conducted (^68^Ga-PSMA CT or MRI), with p=0.127, Gleason (3+4, p=0.99; 4+4, p=0.99; 4+5, p=0.98) or ISUP grade (#2, p=0.99; #3, p=0.99; #4, p=0.98 and #5, p=0.97).

## DISCUSSION

Positron emission tomography-MRI explores MRI multiparametric potential combined with high soft tissue contrast for better tissue characterization, promoting density-based imaging. Furthermore, the use of image sequences of different densities allows visualization of metastases from diverse perspectives and with varying contrasts, potentially increasing the certainty that a positive PET finding is actually present as an anatomical finding.^(10)^

Positron emission tomography has shown good diagnostic properties in prostate cancer since the publication of the first clinical trial with ^68^Ga-PSMA, in 2012.^(11)^Subsequently, the use of PET has been evaluated in different settings, including primary staging and biochemical recurrence.^(18-23)^Luiting et al.^(24)^analyzed nine retrospective and two prospective studies to determine the ability of ^68^Ga-PSMA PET-CT to detect pelvic lymph node metastasis in patients with prostate cancer. The authors studied sensitivity, which ranged between 33.3% and 100%, and specificity, between 80 and 100%, concluding that the test has high specificity with a high positive predictive value, however, with moderate sensitivity, it is still not possible to replace the nodal staging done by lymph node dissection.

Petersen et al.^(25)^ carried out a systematic review aiming to identify diagnostic studies in prostate cancer, comparing preoperative ^68^Ga-PSMA PET-CT or MRI for primary lymph node staging with pathology. A total of 18 clinical trials that included 969 patients, most of them intermediate or high-risk was included. Sixteen studies used ^68^Ga-PSMA; one used ^64^Cu-PSMA and another study used F-18-DCDFPyL. Twelve studies used PET-CT and four used PET-MRI. The diagnostic accuracy varied considerably among them. Sensitivity ranged from 23% to 100%, specificity from 67% to 100%, positive predictive value from 20% to 100%, and negative predictive value from 41% to 100%. The weighted sensitivity and specificity were, respectively, 59% and 93%.^(22)^The differences in findings can be attributed to diverse protocols adopted in the studies.

Recently, Franklin et al.^(26)^ analyzed the ability of ^68^Ga-PSMA PET-CT to predict pathological results of pelvic lymph node involvement. The study included 233 patients, 24.9% of which had a positive histological analysis for lymph node involvement. The sensitivity of ^68^Ga-PSMA PET-CT to predict positive lymph node was 48.3%, and specificity was 92%. The positive predictive value was 66.7% and the negative predictive value was 84.3%.

In another recent study, Klingenberg et al.^(27)^analyzed the accuracy of ^68^Ga-PSMA PET-CT in identifying primary lymph node involvement and distant metastases during the staging of high-risk prostate cancer. In a subgroup analysis of patients who underwent radical prostatectomy with pelvic node dissection, among those in whom lymph node involvement was identified in the pathological analysis, the ^68^Ga-PSMA PET-CT was able to predict lymph node involvement in 11 of 36 cases, with sensitivity of 30.6%, specificity of 96.5%, positive predictive value of 68.8%, and negative predictive value of 83.1%.

In this study, considering preoperative ^68^Ga-PSMA PET-CT and PET-MRI, the overall accuracy, was 85.7%, sensitivity of 50%, and specificity of 97% to identify lymph node involvement compared to pathological analysis. Considering only the unfavorable intermediate and high-risk subgroup, according to D’Amico’s risk classification, accuracy was 81.6%, sensitivity of 50% and specificity of 100%, comparing the analysis of the pathological examination to ^68^Ga-PSMA PET-CT or MRI. Accuracy was 84%, sensitivity of 46.7%, and specificity of 100%, when comparing ^68^Ga-PSMA PET-CT or MRI with the results of pathological examination and postoperative PSA.

Regarding the ability to identify extra prostatic extension and involvement of seminal vesicles, both ^68^Ga-PSMA PET-CT and PET-MRI showed high specificity; however, as expected, because of the principle of the radiological method per se, the CT was much less sensitive than MRI for such diagnoses.

The major limitation of this study was heterogeneity of data in the medical records. In addition, there are intrinsic limitations in the study proposal, since it is a retrospective, single-center, and observational.

## CONCLUSION

^68^Ga-PSMA PET-CT and PET-MRI presented high accuracy in detecting preoperative lymph node involvement and can be very useful tools to indicate the need for extended lymph node dissection during radical prostatectomy.
